# Effects of *Aspergillus niger* and Its Compound Preparations on Methane Emissions and Gastrointestinal Microbiota in Heat-Stressed Holstein Bulls

**DOI:** 10.3390/ani16020154

**Published:** 2026-01-06

**Authors:** Jiangge Wang, Shuaiqi Fu, Xianghui Yin, Shiqin Sun, Tengyun Gao

**Affiliations:** Henan International Joint Laboratory of Nutrition Regulation and Ecological Raising of Domestic Animal, College of Animal Science and Technology, Henan Agricultural University, Zhengzhou 450046, China; wangjge2021@163.com (J.W.); fushuaiqi0371@163.com (S.F.); coolboymommo@outlook.com (X.Y.); 13223861935@163.com (S.S.)

**Keywords:** methane emissions, heat stress, *Aspergillus niger*, oxidative stress, gastrointestinal microbiota

## Abstract

The livestock sector is a major source of methane, a potent greenhouse gas, while heat stress impairs ruminant health. This study evaluated the efficacy of *Aspergillus niger* (AN) and its compound preparation (CP) as feed additives to mitigate methane emissions and heat stress in Holstein bulls. Twenty-four bulls were assigned to a control group (CON), a group supplemented with 6 g/d of AN, and a group with 20 g/d of CP. Methane emissions were measured using the SF_6_ tracer technique. Blood and ruminal fermentation parameters were also analyzed. Results showed that both AN and CP significantly reduced total methane emissions, emissions per unit of body weight, and per unit of dry matter intake compared to CON. Supplemented groups had significantly lower rectal temperatures and higher superoxide dismutase activity, with AN also increasing total antioxidant capacity. AN groups showed increased total volatile fatty acids, acetate, and propionate. Microbiota analysis revealed significant beta-diversity shifts with differential taxon enrichment. In conclusion, *Aspergillus niger* and its compound preparation effectively reduce enteric methane and alleviate heat stress by boosting antioxidant defenses and modulating rumen function, offering a dual-benefit strategy for sustainable ruminant production.

## Simple Summary

In livestock farming, cattle produce methane—a potent greenhouse gas—during digestion, which contributes to climate change. At the same time, hot weather causes heat stress in animals, harming their health and well-being. In this study, we investigated whether adding the natural microorganism *Aspergillus niger* or a compound preparation containing it to the diet of Holstein bulls could help solve these two problems at once. Our results showed that these supplements significantly reduced the methane emissions from the animals. They also helped the bulls cope with heat stress by lowering their body temperature and improving their antioxidant capacity. Furthermore, the additives promoted beneficial digestive processes in the rumen. We conclude that *Aspergillus niger* and its compound preparation are promising, natural feed additives that can make cattle farming more environmentally friendly and help animals better withstand hot summers.

## 1. Introduction

Since the Industrial Revolution, global warming, driven by greenhouse gas emissions, has become a critical global issue [1]. Among the major greenhouse gases, methane (CH_4_) has a global warming potential 25 times greater than that of carbon dioxide (CO_2_), making a particularly significant contribution to climate change [2]. The livestock sector is a major source of anthropogenic CH_4_, accounting for approximately 14.5% of total anthropogenic greenhouse gas emissions globally. A substantial portion of this derives from enteric fermentation in ruminants [3,4]. Simultaneously, the increasing frequency of extreme heat events due to climate warming poses a serious threat to ruminant health through heat stress. This condition triggers a range of adverse effects, including oxidative stress, physiological dysfunction, and reduced production performance and economic returns [5,6,7]. Therefore, the development of green feed additives that can simultaneously mitigate heat stress and reduce enteric CH_4_ emissions in ruminants is crucial for combating climate change and advancing sustainable animal agriculture.

Heat stress affects ruminants through multiple pathways. To reduce metabolic heat production, animals often decrease their dry matter intake (DMI) and dissipate heat through increased respiratory rate and sweating. However, these physiological responses can lead to tissue oxidative damage and compromised antioxidant capacity [7,8,9]. Heat stress also suppresses thyroid hormone secretion (e.g., T3, T4) to lower basal metabolism and triggers the overexpression of heat shock proteins such as HSP70 [10,11,12]. The mechanisms through which heat stress affects methane emissions are complex. Although decreased DMI may lead to reduced CH4 production, heat stress can also promote methane emission by altering animal behavior and the rumen microenvironment. Therefore, this area requires further in-depth investigation [13,14,15].

The rumen is a complex anaerobic ecosystem where dietary nutrients are fermented by microbes, yielding volatile fatty acids (VFA), hydrogen (H_2_), and carbon dioxide (CO_2_). In the predominant hydrogenotrophic pathway, methanogenic archaea use H_2_ to reduce CO_2_, thereby generating CH_4_. This process not only exacerbates the greenhouse effect but also represents a net loss of 2–15% of the animal’s dietary energy [16,17,18]. Current techniques for measuring enteric CH_4_ emissions include respiration chambers, the sulfur hexafluoride (SF_6_) tracer method, and GreenFeed systems. Each method has its applicable scenarios and inherent limitations [19,20,21]. To mitigate CH_4_ emissions, research has primarily focused on nutritional strategies. These include modifying dietary structure, supplementing fats, and using feed additives such as ionophores (e.g., monensin) or chemical inhibitors (e.g., 3-nitrooxypropanol, 3-NOP) [22,23,24]. However, some chemical inhibitors carry potential risks, including chemical residues, the development of microbial resistance, and adverse effects on ruminal fermentation. Consequently, the development of safe and effective natural biological feed additives has emerged as a critical research priority.

Microbial additives, particularly probiotics, have shown considerable promise as alternatives to antibiotics for improving rumen fermentation, enhancing nutrient digestion, and modulating the microbial community [25,26,27]. For example, yeasts can reduce CH_4_ emissions by consuming ruminal oxygen and stimulating acetogenic bacteria to utilize H_2_, thereby directly competing with methanogens for this substrate [28,29]. The filamentous fungus *Aspergillus niger* is widely used in the feed industry. However, its potential dual efficacy in mitigating methane emissions and alleviating heat stress in ruminants has not been thoroughly investigated.

Therefore, this study aimed to evaluate the effects of dietary supplementation with *Aspergillus niger* and its compound preparation on alleviating heat stress and reducing enteric methane emissions in Holstein bulls. We also assessed their impacts on ruminal fermentation parameters and the microbial community. Our findings provide a theoretical foundation for developing novel feed additives that simultaneously confer environmental and health benefits.

## 2. Materials and Methods

### 2.1. Experimental Design

The experiment was carried out between 7 June and 15 July 2023, at the Animal Husbandry Experiment Station of Henan Agricultural University in Qi County. Twenty-four healthy Holstein bulls (approximately 18 months old; initial body weight 350.65 ± 15.26 kg, mean ± SD) were randomly assigned to one of three groups (*n* = 8 per group) in a completely randomized design. The experiment lasted for 31 days, which included a 25-day adaptation period followed by a 6-day data and sample collection period. Enteric methane emission rates were measured from days 26 to 30. Blood and rumen fluid samples were collected on day 31.

### 2.2. Animal Management and Feeding

Before the main experimental period, all bulls underwent a 7-day adaptation to acclimate them to the diets and housing conditions, minimizing stress from regrouping. Daily feed intake and refusals were recorded throughout this phase. The feeding amount was adjusted to ensure that refusals during the subsequent main trial remained at approximately 10% of the feed offered. Throughout the entire experiment, all bulls had ad libitum access to feed and water. They received a total mixed ration (TMR) twice daily at 06:00 and 17:00. The ingredient composition and nutritional profile of the basal TMR are detailed in Table 1 and Table 2.

The bulls were assigned to one of three experimental groups based on the feed additive administered: the control group (CON) received the basal diet only; the *Aspergillus niger* group (AN) received the basal diet supplemented with 6 g/(d·head) of *Aspergillus niger* (spore count ≥ 2 × 10^10^ CFU/g); and the compound probiotics group (CP) received the basal diet supplemented with 20 g/(d·head) of a compound preparation (Ascor Chimici Srl, Bertinoro, Italy). This preparation contained a defined mixture of fermentation products with corn cob powder as the carrier. The active components consisted of *Aspergillus niger* fermentation product, *Aspergillus oryzae* fermentation product, and a yeast-based fraction (comprising *Kluyveromyces marxianus* fermentation product and inactivated dried yeast), formulated in a ratio of 4:4:2 by weight.

### 2.3. Sample Collection and Measurements

#### 2.3.1. Experimental Diet Collection and Analysis

Samples of the total mixed ration (TMR) and the concentrate supplement were collected at the beginning, middle, and end of the trial and stored for subsequent analysis. The samples were oven-dried at 65 °C for 48 h and then cooled to room temperature for 24 h to achieve air-dried samples. The dry matter (DM) content was determined by further drying the air-dried samples at 105 °C for 3 h. All subsequent analyses of feed components are expressed on a DM basis.

The calcium (Ca) content was determined using the EDTA titrimetric method according to the Chinese National Standard GB/T 6436-2018 [30]. Phosphorus (P) content was analyzed by the molybdenum blue spectrophotometric method (GB/T 6437-2018) [31]. The contents of neutral detergent fiber (NDF) and acid detergent fiber (ADF) were analyzed using the methods described by Van Soest et al. [32]. The crude fat (EE) and crude protein (CP) contents were determined according to the standard methods of AOAC (method numbers 920.39 and 984.13, respectively) [33,34].

#### 2.3.2. Determination of Temperature-Humidity Index, Rectal Temperature, and Respiratory Rate

Three temperature and humidity data loggers (Model VCH6003, ONABEE, Shenzhen, China) were positioned at both ends and the center of the barn at a height of 1.5 m to continuously monitor ambient temperature and relative humidity. The temperature-humidity index (THI) was calculated according to the following formula:

$$THI\left(\%\right)=\left(1.8\times T+32\right)-0.55\times (1-RH)\times (1.8\times T-26)$$

In the formula, THI represents the temperature-humidity index, T denotes the temperature (°C), and RH is the relative humidity (%).

During the adaptation period (days 1–25), rectal temperature and respiratory rate were monitored daily between 13:00 and 15:00. Rectal temperature was measured with a digital veterinary thermometer (Model SAT-1, Shandong Shangnong Electronic Technology Co., Ltd., Jinan, China). Respiratory rate was determined by counting flank movements over a 60 s period when the animal was at rest. All measurements were taken in triplicate, and the mean value was recorded for analysis.

#### 2.3.3. Blood Sample Collection and Biochemical Analysis

On the final experimental day (day 31), blood samples were collected from the coccygeal vein before morning feeding using K2EDTA(Becton, Dickinson and Company, Shanghai, China) vacuum tubes. The blood samples were centrifuged at 3000× *g* for 10 min to separate plasma, which was then aliquoted and stored at –20 °C until further analysis.

The serum concentrations of immunoglobulins (IgA, IgG, IgM), thyroid hormones (T3, T4), cortisol, and heat shock proteins (HSP70, HSP90) were measured using commercial enzyme-linked immunosorbent assay (ELISA) kits(Nanjing Jiancheng Bioengineering Institute, Nanjing, China). The activity of total antioxidant capacity (T-AOC), superoxide dismutase (SOD), and catalase (CAT), along with the concentration of malondialdehyde (MDA), was determined using spectrophotometric commercial assay kits(Nanjing Jiancheng Bioengineering Institute, Nanjing, China) according to the manufacturers’ instructions.

#### 2.3.4. Collection and Analysis of Rumen Fluid and Fecal Samples

On the final experimental day prior to morning feeding, rumen fluid samples were collected from all bulls using a rumen tube (oro-ruminal sampling method). The first few milliliters were discarded to eliminate contamination from saliva and feed, after which subsequent fluid was collected. The pH of the fresh rumen fluid was immediately measured with a portable pH meter (Model HD-Y05, Shandong Hualde Electronic Technology Co., Ltd., Jinan, China). Samples were then aliquoted, flash-frozen in liquid nitrogen for microbial community analysis, and stored at –40 °C for subsequent determination of rumen fermentation parameters.

Fecal samples were collected directly from the rectum. Before sampling, the perianal area was cleaned. Samples were immediately flash-frozen in liquid nitrogen and then stored at −80 °C until microbial DNA extraction and analysis.

Rumen ammonia nitrogen (NH_3_-N) concentration was determined using the standard phenol-hypochlorite colorimetric method, with absorbance measured at 630 nm. The concentrations of individual volatile fatty acids (VFA) were quantified by gas chromatography (GC; model GCI120, Shimadzu, Kyoto, Japan) equipped with a flame ionization detector and a capillary column (TP-Porapak Q, 15 m × 320 μm × 5 μm).

#### 2.3.5. Permeation Tube Preparation

The sulfur hexaluoride (SF_6_) tracer technique was used to measure enteric methane emissions from the bulls. Empty permeation tubes were first weighed to obtain the initial mass (W_0_) and then preheated overnight in a 39 °C incubator. After cooling in liquid nitrogen, the tubes were filled with ≥0.8 g of SF_6_ gas and hermetically sealed. The sealed tubes were re-weighed and allowed to equilibrate to room temperature before being stabilized again at 39 °C. The stabilized mass (W_1_) was then measured using an analytical balance (FA2004G, Shanghai Huachao Electric Co., Ltd., Shanghai, China). Based on the calculated release rates, 24 tubes exhibiting stable permeation were selected for the experiment. The mass of SF_6_ loaded into each tube was calculated as follows:

$$W_{{SF}_{6}}=W_{1}-W_{0}$$

In the formula, W_SF6_ represents the mass (g) of SF_6_ filled into the permeation tube; W_1_ denotes the total mass (g) of the permeation tube and the sealed SF_6_; and W_0_ is the mass (g) of the empty permeation tube.

SF_6_ Permeation Tube Preparation and Release Rate Determination: The SF_6_ permeation tubes were incubated at 39 °C in sealed containers purged with nitrogen to simulate anaerobic ruminal conditions. The tubes were weighed every third day at 15:00, with a total of 15–20 repeated measurements. Data from the initial 20-day period of unstable release were excluded from the analysis. A linear regression of SF_6_ mass against time was established using the subsequent stable data, and the slope of this regression was defined as the permeation rate. The specific permeation rates and corresponding regression equations for each tube are presented in Supplementary Table S1.

#### 2.3.6. Collection and Measurement of Gastrointestinal Methane

Enteric CH_4_ Collection Using the SF_6_ Tracer Technique: Enteric methane (CH_4_) emissions were quantified using the sulfur hexafluoride (SF_6_) tracer technique with a 12 h fixed-interval sampling protocol. On day 12 of the trial, permeation tubes with predetermined release rates were placed into the rumen of each bull using a balling gun. The animals were observed for 5 min after dosing to ensure no regurgitation occurred. Gas sampling was conducted from days 26 to 30. Each day between 07:00 and 19:00, approximately 100 mL of gas was collected hourly using a disposable syringe from multiple sites around the animal’s muzzle and transferred into a pre-evacuated 2 L gas sampling bag (Model MBT11, Dalian Haide Technology Co., Ltd., Dalian, China). The collected gas in each bag was thoroughly mixed at the end of the daily collection period. Concentrations of SF_6_ and CH_4_ were analyzed within 72 h after sampling.

The methane emissions were measured over a continuous 6-day period (days 26–30) using the sulfur hexafluoride (SF_6_) tracer technique. This sampling duration aligns with the established protocol for the SF_6_ method, which typically employs integrated breath sampling over 5–7 days to obtain representative daily emission estimates [35]. Under controlled feeding conditions, a 5-day sampling window has been shown to yield consistent CH_4_ emission values comparable to longer sampling periods [35]. While rumen fermentation parameters can exhibit longer-term dynamics after dietary changes [36], methane emissions are often measured over short-term periods in nutritional studies to detect treatment differences. Thus, the 6-day measurement period was selected to ensure methodological rigor while capturing meaningful treatment effects on methane production.

The concentration of SF_6_ was analyzed using a gas chromatograph (GC-4000A, Beijing East & West Analysis Instrument Co., Ltd., Beijing, China) equipped with an electron capture detector (ECD). The analysis employed a 5Å molecular sieve column maintained at 50 °C, with the injector and detector temperatures set at 100 °C and 250 °C, respectively. A 1 mL gas sample was injected using a 0.15 ppb SF_6_ standard. High-purity nitrogen (99.99%) was used as the carrier gas at a constant flow rate of 20 mL/min, and quantification was based on the external standard method.

The CH_4_ concentration was analyzed using a gas chromatograph (GC1120, Shanghai Shimadzu Hengping Scientific Instrument Co., Ltd., Shanghai, China) equipped with a flame ionization detector (FID). The separation was performed on a TP-Porapak Q capillary column (15 m × 320 μm × 5 μm) with an oven temperature of 55 °C, a detector temperature of 200 °C, and an injector temperature of 150 °C. High-purity nitrogen was used as the carrier gas at a flow rate of 10 mL·min^−1^. A standard curve was established using a series of SF_6_ standard gases (concentration range: 4.9 to 20.0 × 10^−6^ ppm), and the CH_4_ concentration in the samples was calculated via the external standard method.

The calculation formula for the gastrointestinal CH_4_ emission rate in Holstein bulls is as follows:

$$R_{{CH}_{4}}=\frac{R_{{SF}_{6}}}{6.518}\times \frac{\left[{CH}_{4}\right]}{\left[{SF}_{6}\right]}\times 10^{3}$$

In the formula, R_CH4_ represents the gastrointestinal CH_4_ emission rate (L/d); R_SF6_ denotes the permeation rate of SF_6_ from the tube (mg/d); 6.518 is the density of SF_6_ (kg/m^3^); [CH_4_] is the measured concentration of CH_4_ in the gas sample (ppm, ×10^−6^); and [SF_6_] is the measured concentration of SF_6_ in the gas sample (ppt, ×10^−12^).

### 2.4. Microbial Community Analysis of Rumen Fluid and Fecal Samples

The ruminal and fecal microbial communities were characterized by sequencing the 16S rRNA gene, with all laboratory procedures performed by Shanghai Yuanxin Biomedical Technology Co., Ltd.(Shanghai, China) Genomic DNA was extracted and its quality was verified by 1% agarose gel electrophoresis. The hypervariable V3–V4 regions were amplified in triplicate on an ABI GeneAmp^®^ 9700 thermal cycler using the barcoded universal primers 338F/806R and TransStart FastPfu DNA Polymerase (AP221-02, TransGen, Beijing, China). The pooled PCR products for each sample were examined on a 2% agarose gel and then purified with the AxyPrep DNA Gel Extraction Kit (Axygen, Shanghai, China). The purified amplicons were eluted in Tris-HCl buffer, quantified fluorometrically (QuantiFluor™-ST, Promega Corpation, Madison, Wisconsin, USA), and pooled in equimolar ratios to construct a sequencing library. The library was prepared according to the standard Illumina protocol, which involved the ligation of index adapters, fragment size selection with magnetic beads, and PCR enrichment. Finally, the library was denatured and subjected to paired-end sequencing (2 × 250 bp) on an Illumina PE250 platform.

### 2.5. Data Processing and Statistical Analysis

All data were initially organized using Microsoft Excel 2016. One-way analysis of variance (ANOVA) was performed with SPSS 26.0, and significant differences among groups were assessed using Duncan’s multiple range test. Data are presented as the mean ± standard error of the mean (SEM), with statistical significance defined at *p* < 0.05.

For microbial community analysis, rarefaction curves were generated in mothur and visualized using R. Principal coordinate analysis (PCoA) based on Unifrac distances was also performed in R to evaluate beta diversity. To identify microbial taxa with significant differences in abundance among groups, Linear Discriminant Analysis Effect Size (LEfSe) was applied, which combines linear discriminant analysis (LDA) with effect size measurements to detect features that most likely explain differences between experimental conditions.

## 3. Results

### 3.1. Barn Temperature and Humidity

Throughout the experimental period, the barn environment was characterized by persistently high temperature and humidity. The temperature–humidity index (THI) consistently exceeded the heat stress threshold of 68, ranging from 73.81 to 85.19 with a mean of 81.2 (Supplementary Figure S1). These data confirm that the bulls experienced continuous heat stress during the study.

### 3.2. Effects of Aspergillus niger and the Compound Preparation on Respiratory Rate and Rectal Temperature in Holstein Bulls

Dietary supplementation with *Aspergillus niger* (AN) or the compound preparation (CP) significantly influenced the physiological responses of heat-stressed bulls (Table 3). Both the AN and CP groups exhibited a significantly lower rectal temperature compared to the CON group (*p* < 0.05). In contrast, the respiratory rate did not differ significantly among the three groups (*p* > 0.05).

### 3.3. Effects of Aspergillus niger and the Compound Preparation on Blood Biochemical Indices in Holstein Bulls

#### 3.3.1. Plasma Parameters Unaffected by Dietary Supplementation

Dietary supplementation with *Aspergillus niger* or the compound preparation did not induce significant changes in a suite of systemic plasma biomarkers compared to the control (CON) group (all *p* > 0.05; Supplementary Table S2). This included heat shock proteins (HSP70 and HSP90), key metabolic and stress hormones (triiodothyronine—T3, thyroxine—T4, cortisol, and the T3/T4 ratio), as well as immunoglobulins (IgG, IgA, and IgM).

#### 3.3.2. Effects on Plasma Antioxidant Capacity

In contrast, the supplements significantly modulated the systemic antioxidant status of the bulls (Table 4). Superoxide dismutase (SOD) activity was markedly increased in both the AN and CP groups relative to the CON group (*p* < 0.05). Furthermore, total antioxidant capacity (T-AOC) was specifically enhanced in the AN group compared to both the CON and CP groups (*p* < 0.05). Catalase (CAT) activity and malondialdehyde (MDA) levels, however, remained comparable across all three groups (*p* > 0.05).

### 3.4. Effects of Aspergillus niger and the Compound Preparation on Rumen Fermentation Parameters

Ruminal fermentation parameters, specifically the volatile fatty acid (VFA) profile, are summarized in Table 5. The AN group exhibited markedly increased concentrations of total VFA, acetate, propionate, isobutyrate, and valerate compared to both the CON and CP groups (*p* < 0.05). In contrast, the CP group showed no significant differences from the CON group in these parameters (*p* > 0.05). Furthermore, the concentrations of butyrate and isovalerate, as well as the acetate-to-propionate ratio, remained comparable across all three groups (*p* > 0.05).

**Figure 1:**
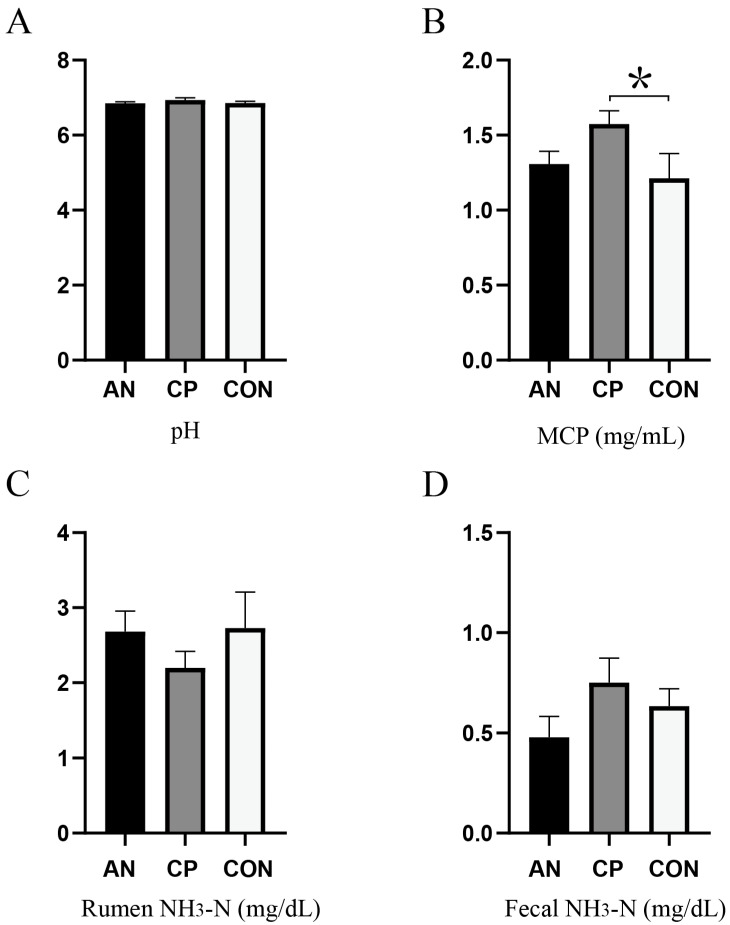
Effects of *Aspergillus niger* and its compound preparation on rumen fermentation and digestive parameters. (**A**) Ruminal pH; (**B**) Microbial crude protein (MCP); (**C**) Ruminal ammonia nitrogen (NH_3_-N); (**D**) Fecal ammonia nitrogen (NH_3_-N) * indicates a significant difference (*p* < 0.05).

Ruminal fermentation parameters and fecal NH_3_-N levels are summarized in Figure 1. No significant differences were observed in ruminal pH, ruminal NH_3_-N, or fecal NH_3_-N concentrations among the three groups (*p* > 0.05). In terms of microbial crude protein (MCP), the CP group exhibited a significantly higher level than the CON group (*p* < 0.05), while the MCP level in the AN group was not significantly different from that of the CON group (*p* > 0.05).

### 3.5. Effects of Aspergillus niger and the Compound Preparation on Intestinal Methane Emissions in Holstein Bulls

The effects of dietary supplements on enteric methane emissions are summarized in Table 6. Both the AN and CP groups significantly reduced total methane emissions, as well as methane yield per unit of body weight and dry matter intake (DMI), compared to the CON group (*p* < 0.05). No significant differences in these emission metrics were detected between the AN and CP groups (*p* > 0.05). Overall, dietary supplementation with *Aspergillus niger* and the compound preparation reduced total enteric methane emissions by 41.47% and 53.65%, respectively.

### 3.6. Effects of Aspergillus niger and the Compound Preparation on Rumen Microbial Diversity in Holstein Bulls

#### 3.6.1. Assessment of Rumen Microbial Diversity

The alpha diversity of the rumen microbiota, assessed by indices including Ace, Chao, Shannon, and Simpson, did not differ significantly among the CON, AN, and CP groups (*p* > 0.05; Supplementary Table S3), indicating that dietary supplementation did not markedly alter the overall richness or evenness of the microbial community. The library coverage for each group exceeded 98.7%, confirming that the sequencing depth was sufficient to reliably capture the diversity present. Detailed sequencing metrics (e.g., reads, OTU numbers) are provided in Supplementary Table S3.

#### 3.6.2. Analysis of Rumen Microbial Community Structure

**Figure 2:**
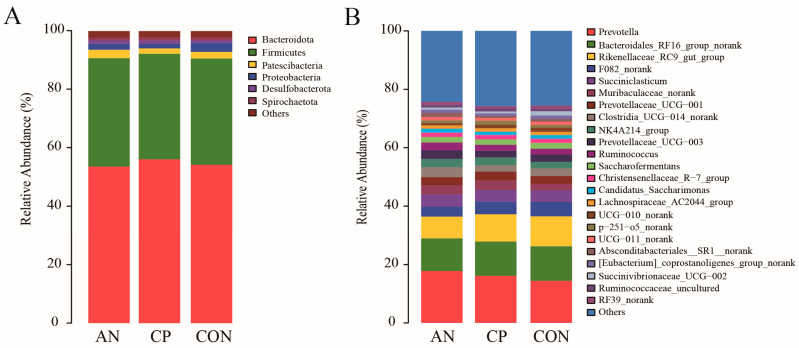
Rumen microbial community structure. **Note:** Panel (**A**) displays the taxonomic composition at the *phylum* level; Panel (**B**) shows the composition at the *genus* level.

The rumen microbial community structure is depicted in Figure 2 (A: phylum level; B: genus level). At the phylum level, the microbiota was primarily composed of *Bacteroidota* and *Firmicutes*, which together constituted the vast majority of the sequences. The relative abundance of *Bacteroidota* was 54.28%, 53.67%, and 56.13% in the CON, AN, and CP groups, respectively. *Firmicutes* accounted for 36.38%, 37.11%, and 36.14% in the same groups. The combined dominance of these two phyla was consistent across all dietary treatments.

At the genus level, taxonomic analysis identified a complex consortium of bacteria in the rumen microbiota across all experimental groups. Key genera detected included *Prevotella* (and related taxa *Prevotellaceae_UCG-001* and *Prevotellaceae_UCG-003*), *Bacteroidales_RF16_group*, *Rikenellaceae_RC9*_*gut_group*, *Succiniclasticum*, *uncultured Muribaculaceae*, *Clostridia_UCG-014*, *Ruminococcus*, *Lachnospiraceae_AC2044_group*, and *Succinivibrionaceae*_*UCG-002*, among others. Across all treatments, the community was consistently dominated by three core genera: *Prevotella*, *Bacteroidales*_*RF16_group*, and *Rikenellaceae*_*RC9_gut_group* (Figure 2B). Their relative abundances varied by dietary treatment: in the CON group, they constituted 14.42%, 11.85%, and 10.25% of the rumen microbiota, respectively; in the AN group, 17.77%, 11.21%, and 7.46%; and in the CP group, 16.15%, 11.76%, and 9.31%.

#### 3.6.3. Analysis of Rumen Microbial β-Diversity

**Figure 3:**
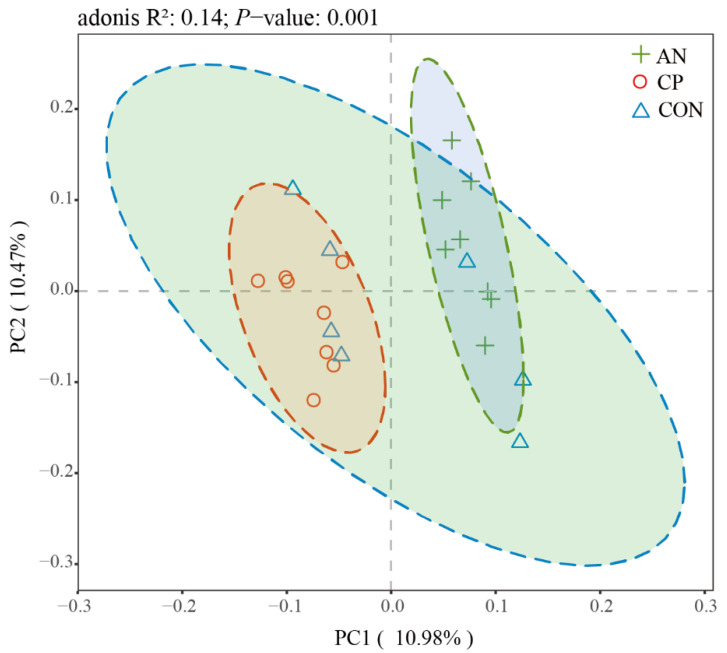
Rumen microbial PCoA (Unifrac analysis). **Note**: The dashed circles in different colors outline the approximate boundaries of the microbial communities for each experimental group: control (CON, blue), *Aspergillus niger* (AN, green), and compound preparation (CP, red).

The beta diversity of the rumen microbiota, assessed by principal coordinates analysis (PCoA) based on UniFrac distances, is shown in Figure 3. The first two principal coordinates (PCo1 and PCo2) explained 10.98% and 10.47% of the total variance, respectively, resulting in a cumulative contribution of 21.45%. This level of variance explanation is sufficient to reveal structural differences in the microbial communities among the groups.

The PCoA plot revealed distinct clustering patterns among the groups. Samples from the CP group (red) clustered primarily in the lower-left quadrant, while those from the AN group (green) aggregated in the upper-right quadrant, showing a clear separation between these two treatments. In contrast, samples from the CON group (blue) were dispersed in the intermediate space between the AN and CP clusters.

Permutational multivariate analysis of variance (Adonis) confirmed that dietary supplementation significantly altered the rumen microbial β-diversity (R^2^ = 0.14, *p* = 0.001), explaining approximately 14% of the total variance in community structure among the CON, AN, and CP groups.

#### 3.6.4. Analysis of Differential Rumen Microbial Taxa

**Figure 4:**
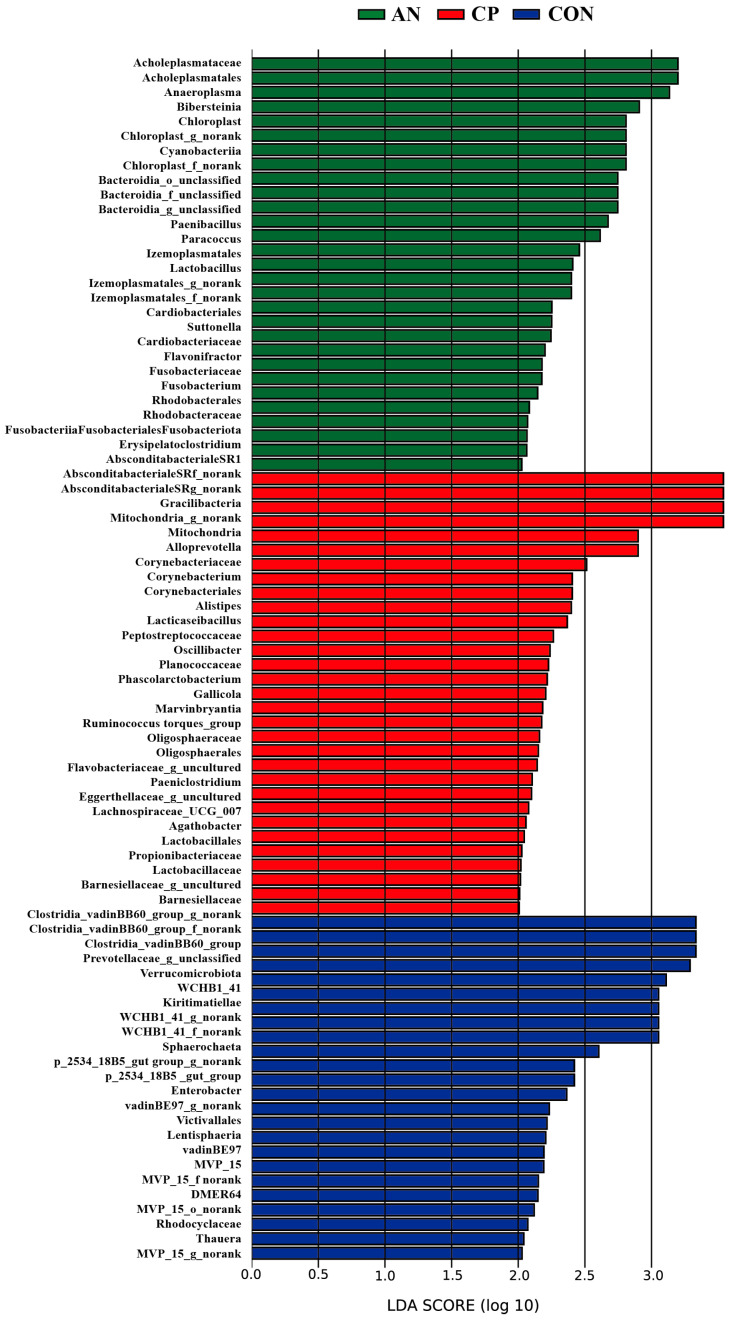
Histogram of LDA distribution of rumen differential microorganisms.

The Linear Discriminant Analysis Effect Size (LEfSe) identified distinct microbial taxa enriched in each group (LDA score > 2.0), as shown in Figure 4. In total, 24 taxa were significantly enriched in the CON group. Among these, nine taxa exhibited particularly strong effects (LDA > 3.0), including *Verrucomicrobiota*, *Kiritimatiellae*, *WCHB1_41*, and several unclassified members of the *Clostridia_vadinBB60_group* and *Prevotellaceae*.

Distinct microbial signatures were also identified in the supplemented groups. The AN group exhibited significant enrichment of 31 microbial taxa, four of which showed strong effects (LDA > 3.0), including the order *Absconditabacteriales_SR1* and its associated family and genus (*Absconditabacteriales_SR1_f_norank*, *Absconditabacteriales_SR1_g_norank*), as well as the phylum *Gracilibacteria*.

Similarly, the CP group was enriched with 29 taxa, three of which had high LDA scores (>3.0), namely the order *Acholeplasmatales*, its family *Acholeplasmataceae*, and the genus *Anaeroplasma*.

### 3.7. Effects of Aspergillus niger and the Compound Preparation on Fecal Microbial Diversity in Holstein Bulls

#### 3.7.1. Assessment of Fecal Microbial Diversity

The alpha diversity of the fecal microbiota, as indicated by the Ace, Chao, Shannon, and Simpson indices, showed no significant differences among the CON, AN, and CP groups (*p* > 0.05; Supplementary Table S3). This result suggests that dietary supplementation did not substantially alter the overall diversity structure of the fecal microbial community. High library coverage (>99.5%) for all samples confirmed the adequacy of sequencing depth (Supplementary Table S3).

#### 3.7.2. Analysis of Fecal Microbial Community Structure

**Figure 5:**
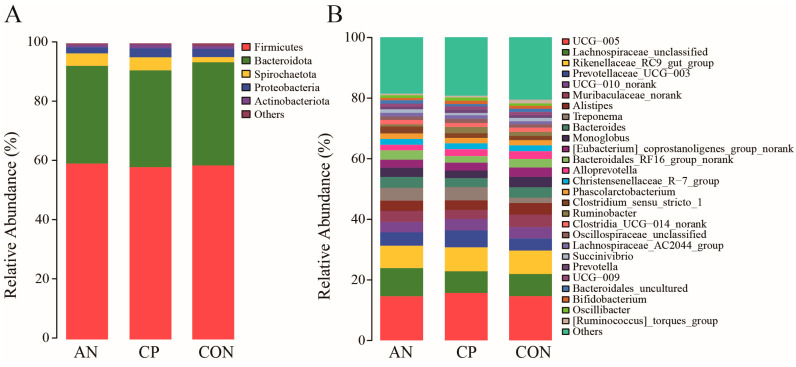
Fecal microbial community structure. **Note:** Panel (**A**) displays the taxonomic composition at the *phylum* level; Panel (**B**) shows the composition at the *genus* level.

The fecal microbiota at the phylum level was predominantly composed of *Firmicutes* and *Bacteroidota* across all experimental groups (Figure 5A). The relative abundance of *Firmicutes* was 58.75%, 59.38%, and 58.18% in the CON, AN, and CP groups, respectively, while *Bacteroidota* accounted for 34.86%, 33.02%, and 32.69%. Other phyla consistently present included *Spirochaetota*, *Proteobacteria*, and *Actinobacteriota*, though their abundances remained comparatively low.

At the genus level, more than ten genera were commonly detected across the three groups (Figure 5B). Among these, *Ruminococcaceae_UCG-005*, *Lachnospiraceae_unclassified*, *Rikenellaceae_RC9_gut_group*, and *Prevotellaceae_UCG-003* were identified as the dominant genera. Their relative abundances varied among groups: *Ruminococcaceae_UCG-005* accounted for 14.62%, 14.57%, and 15.68% in the CON, AN, and CP groups, respectively; *Lachnospiraceae_unclassified* for 7.28%, 9.29%, and 7.13%; *Rikenellaceae_RC9_gut_group* for 7.76%, 7.38%, and 7.92%; and *Prevotellaceae_UCG-003* for 3.93%, 4.44%, and 5.53%.

#### 3.7.3. Analysis of Fecal Microbial β-Diversity

**Figure 6:**
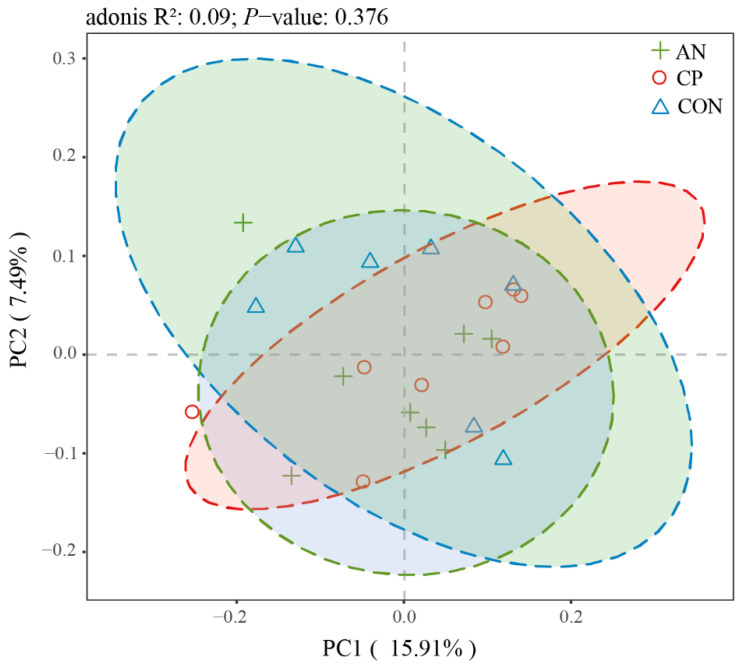
Fecal microbial PCoA (Unifrac analysis). **Note:** The dashed circles in different colors outline the approximate boundaries of the microbial communities for each experimental group: control (CON, blue), *Aspergillus niger* (AN, green), and compound preparation (CP, red).

The beta diversity of the fecal microbiota, as assessed by principal coordinates analysis (PCoA) based on UniFrac distances, is displayed in Figure 6. The first two principal coordinates (PCo1 and PCo2) explained 15.91% and 7.49% of the total variance, respectively, cumulatively accounting for 23.4% of the observed structural variation. This result supports the use of these axes to represent key compositional differences in the fecal microbial communities among the groups.

Analysis of the fecal microbiota beta diversity revealed no significant structural shifts in response to dietary supplementation. As shown in the PCoA plot (Figure 6), samples from the CON, AN, and CP groups exhibited substantial overlap with no clear separation. This observation was statistically confirmed by permutational multivariate analysis of variance (Adonis), which indicated no significant differences in community structure among the groups (R^2^ = 0.04, *p* = 0.376).

#### 3.7.4. Analysis of Differential Fecal Microbial Taxa

**Figure 7:**
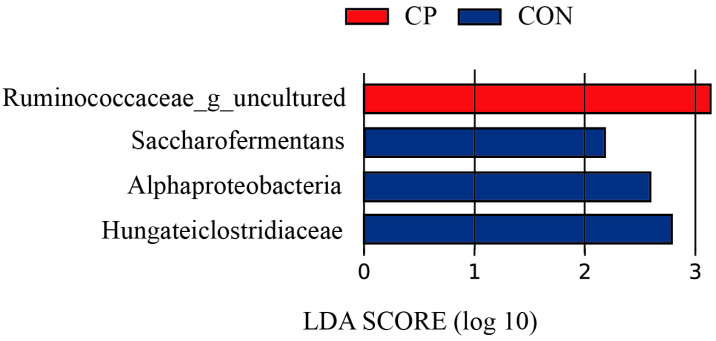
Histogram of LDA distribution of fecal differential microorganisms.

Linear discriminant analysis Effect Size (LEfSe) revealed distinct fecal microbial taxa enriched in specific groups (LDA score > 2.0, Figure 7). Three taxa were significantly enriched in the CON group: *Alphaproteobacteria*, *Saccharofermentans*, and *Hungateiclostridiaceae*. In the CP group, only one taxon, *Ruminococcaceae_g_uncultured*, was significantly enriched. In contrast, no microbial taxa were found to be significantly enriched in the AN group.

## 4. Discussion

Under high temperature and humidity conditions, heat stress induces a range of physiological adjustments in animals to maintain thermal balance, among which changes in respiratory rate and rectal temperature are the most evident [37]. In cattle, heat stress is defined as a temperature–humidity index (THI) exceeding 68, with categories ranging from mild (68 < THI < 72) and moderate (72 < THI < 80) to severe (80 < THI < 90) [37]. Throughout the present experiment, the recorded THI varied between 73.81 and 85.19, indicating that the bulls were subjected to persistent, moderate-to-severe heat stress. Under such conditions, the capacity for heat dissipation is compromised. To stabilize core body temperature, cattle undergo physiological adaptations such as increased sweating, elevated respiratory and heart rates, and a rise in body temperature [6]. Previous studies have confirmed significant increases in both rectal temperature and respiratory rate in heat-stressed beef cattle [38]. Moreover, dietary interventions, such as supplementation with fermented herbal tea residues containing compound probiotics, have been reported to alleviate heat stress by reducing rectal temperature [39]. In line with these findings, the present study demonstrated that dietary supplementation with either *Aspergillus niger* or its compound preparation significantly reduced rectal temperature in Holstein bulls. These results suggest that both additives can effectively attenuate heat stress-induced hyperthermia, thereby mitigating its adverse physiological consequences.

The thermoneutral zone for most livestock species ranges from 16 °C to 25 °C. Exposure to elevated temperatures promotes the generation of reactive oxygen species (ROS), leading to oxidative damage in lipids, proteins, and DNA [40,41]. As a defense mechanism, organisms under stress activate the synthesis of heat shock proteins (HSPs) [42]. Functioning as molecular chaperones, HSPs bind to denatured proteins and prevent their irreversible aggregation, thereby serving as both protective agents and biomarkers of heat stress severity [43]. For instance, Pearce et al. [44] reported elevated HSP70 mRNA expression in muscle tissues of animals under sustained heat stress. In the present study, plasma HSP70 levels were significantly lower in the CP group than in the CON group. Although the reductions in HSP70 and HSP90 in the AN group and HSP90 in the CP group did not reach statistical significance, their values consistently trended downward. These findings suggest that dietary supplementation with *Aspergillus niger* or its compound preparation may attenuate heat stress by modulating HSP expression in Holstein bulls. Nevertheless, the precise molecular mechanisms involved warrant further investigation.

Reactive oxygen species (ROS) are natural byproducts of cellular metabolism; however, their excessive accumulation under heat stress can overwhelm the endogenous antioxidant defense system, resulting in systemic oxidative damage [45,46,47]. In the present study, dietary supplementation with *Aspergillus niger* (AN) significantly enhanced the plasma total antioxidant capacity (T-AOC) compared to both the CON and CP groups, whereas the CP group showed no significant difference from the CON group. These results demonstrate that *Aspergillus niger* alone effectively augments the systemic antioxidant capacity in Holstein bulls under heat stress, which may contribute to the alleviation of heat stress-induced oxidative damage.

Superoxide dismutase (SOD) serves as a critical primary defense enzyme that detoxifies superoxide radicals, protects mitochondrial membrane integrity, and prevents oxidative damage [48]. Seasonal variations demonstrate the importance of this antioxidant defense, as evidenced by Rathwa et al. [49] who reported higher plasma SOD activity in indigenous Indian sheep during summer compared to winter months. Furthermore, under heat stress conditions, both serum T-AOC and SOD activity significantly decrease in lactating dairy cows, and this reduction in antioxidant capacity correlates with decreased milk production [50].

The present study demonstrated that dietary supplementation with either *Aspergillus niger* or the compound preparation notably elevated plasma superoxide dismutase (SOD) activity. Furthermore, supplementation with *Aspergillus niger* alone specifically enhanced the total antioxidant capacity (T-AOC). These collective improvements in key antioxidant parameters contributed to an enhanced overall antioxidant defense in Holstein bulls, thereby reducing the risk of systemic oxidative damage induced by heat stress.

Thyroid hormones, notably T3 and T4, play essential roles in regulating metabolic activity, growth performance, and thermogenesis in animals, making them valuable indicators of heat tolerance in livestock [51]. Heat stress disrupts the hypothalamic–pituitary-thyroid axis, suppressing the secretion of thyrotropin-releasing hormone and subsequently reducing circulating T3 and T4 levels, which in turn lowers basal metabolic rate [52]. In the present study, although the increases in plasma T3 and T4 levels in the AN and CP groups were not statistically significant, both groups exhibited numerically higher values than the CON group. This trend suggests that *Aspergillus niger* and its compound preparation may help modulate body temperature and energy metabolism in Holstein bulls by supporting thyroid hormone homeostasis, thereby contributing to thermal balance under heat stress. In contrast, plasma cortisol levels did not differ significantly among the three groups, indicating that the supplements did not markedly influence this stress-related biomarker under the present experimental conditions. The underlying mechanisms governing these responses remain to be fully elucidated and warrant further investigation.

The assessment of humoral immunity revealed that plasma levels of IgG, IgA, and IgM remained unchanged across the experimental groups (Table S2). While heat stress is known to potentially suppress immune function, our results suggest that supplementation with *Aspergillus niger* or its compound preparation did not exert a direct modulating effect on these specific systemic antibody levels within the trial period. Similar findings have been reported in other ruminant studies where certain feed additives improved production or environmental parameters without significantly altering circulating immunoglobulin concentrations [53]. Therefore, the primary benefits observed in this study—reduced methane emissions and alleviated heat stress—appear to be more closely linked to enhanced antioxidant capacity and modified rumen function rather than to a systemic humoral immune response.

Dietary supplementation with microbial additives contributes to the maintenance of ruminal pH, thereby helping prevent ruminal acidosis while promoting microbial balance and homeostasis [54]. Previous studies have indicated that heat stress can alter the concentrations of ruminal volatile fatty acids (VFA) through changes in dry matter intake [55,56]. In the present study, ruminal pH did not differ among the CON, AN, and CP groups, indicating that supplementation with *Aspergillus niger* or the compound preparation did not disrupt normal ruminal acidity and may help maintain a stable ruminal environment. The absorption and transport of VFA are closely linked to the structure of the ruminal epithelium, and a substantial portion of the energy required for ruminal digestion is derived from VFA metabolism [57]. In this experiment, the AN group showed significantly higher concentrations of total VFA, acetate, propionate, isobutyrate, and valerate than both the CON and CP groups. This shift may be attributed to microbial population changes induced by *Aspergillus niger*, leading to enhanced feed fermentation and an altered VFA profile. The elevated VFA levels suggest improved metabolic activity in the rumen, generating more metabolizable energy that could partially compensate for the reduced dry matter intake typically associated with heat stress.

Probiotic strains vary in their efficacy for mitigating enteric methane emissions. For example, *Acetobacter* strain GA03 exhibits stronger inhibitory effects on methanogenesis than other strains [58]. In general, probiotics can reduce methane production by modulating ruminal microbial activity while enhancing fermentation efficiency without adverse effects on animal health [59]. Dietary supplementation with lactic acid bacteria not only decreases methane emissions per unit of volatile fatty acids produced but also improves silage fermentation quality and fiber digestibility [60]. Similarly, *Bacillus licheniformis* has been reported to suppress methane generation while promoting the utilization of dietary energy and protein [61]. In the present study, both the AN and CP groups exhibited significant reductions in total methane emission, methane yield per unit of body weight, and methane yield per unit of dry matter intake relative to the CON group. These results demonstrate the potential of *Aspergillus niger* and its compound preparation as effective anti-methanogenic feed additives for Holstein bulls. This finding is consistent with the evaluation framework proposed by Hristov et al. [62], which emphasizes the importance of modulating rumen fermentation and employing the above metrics to assess the mitigation efficacy of candidate feed additives.

Dietary supplementation with *Aspergillus niger* or its compound preparation delivered dual benefits: it significantly reduced enteric methane emissions (in terms of total output, yield per unit body weight, and yield per unit DMI) and alleviated heat stress in Holstein bulls. The mitigation of heat stress was evidenced by a lower rectal temperature, a numerical reduction in plasma HSP70 levels (though not statistically significant), and an enhanced antioxidant defense system. Specifically, superoxide dismutase (SOD) activity was significantly increased in both supplemented groups, and total antioxidant capacity (T-AOC) was significantly elevated in the *Aspergillus niger* group, collectively contributing to reduced oxidative damage. Furthermore, dietary supplementation with *Aspergillus niger* enhanced ruminal fermentation by increasing the concentrations of total volatile fatty acids (VFA), acetate, propionate, isobutyrate, and valerate. This metabolic shift promotes nutrient absorption and utilization, thereby helping to compensate for the reduced nutrient supply resulting from heat stress-induced decreases in dry matter intake.

Ruminants digest feed through a sequential process involving the rumen, reticulum, omasum, and finally the abomasum. The rumen hosts a vast and complex microbial ecosystem that enables the breakdown of fibrous materials and the conversion of non-protein nitrogen into microbial protein [63]. This microbial community is influenced by multiple factors—including diet, host physiology, and early-life exposure—with dietary intervention being the most direct and significant driver of microbial population shifts [64]. In a comprehensive review, Newbold [63] outlined the potential benefits and limitations of various dietary additives for ruminants. Following the widespread ban on antibiotic growth promoters, research efforts have increasingly focused on alternative strategies, particularly probiotics and plant-derived extracts.

Analysis of alpha diversity indices showed that the Ace index tended to be lower in the AN group than in the CON group, whereas no significant differences were observed among the three groups for the Chao, Shannon, Simpson, or Coverage indices. This implies that supplementation with *Aspergillus niger* may have selectively influenced certain aspects of microbial richness without broadly altering overall diversity. In terms of beta diversity, although no clear separation was detected among the CON, AN, and CP groups in the PCoA, cluster analysis revealed distinct clustering between the AN and CP groups. This suggests that while the overall rumen microbial structure remained similar to the control, the two additives induced specific compositional shifts that differentiated their microbial profiles from each other. These observations align with previous studies reporting that probiotic supplementation in beef cattle often does not affect alpha diversity indices but can lead to significant separation in microbial clustering between treatments [65].

Analysis of fecal microbial communities revealed no significant differences in either alpha or beta diversity among the CON, AN, and CP groups. This indicates that the effects of *Aspergillus niger* and the compound preparation were mainly localized within the gastrointestinal tract, with limited impact on the distal gut microbiota. Throughout the experimental period, all animals remained healthy without exhibiting abnormal behaviors or clinical symptoms. These observations confirm that dietary supplementation with these additives did not adversely affect animal health or welfare under the conditions of this study.

The rumen microbiota is integral to feed digestion and nutrient metabolism in ruminants. In this study, *Bacteroidota* and *Firmicutes* were identified as the dominant phyla in both the ruminal and fecal microbiota across all experimental groups. Similarly, at the genus level, the composition of predominant bacterial groups remained largely consistent among the CON, AN, and CP groups, indicating a high degree of structural stability in the core microbiota. These results suggest that dietary supplementation with *Aspergillus niger* or the compound preparation did not substantially alter the dominant microbial communities in either the rumen or feces. This finding aligns with previous ruminant studies, which have consistently reported *Firmicutes* and *Bacteroidota* as the two most abundant bacterial phyla in the rumen ecosystem [66,67]. *Firmicutes*, predominantly Gram-positive, include many taxa involved in fiber degradation and lactate production [8,68,69], whereas *Bacteroidota* contribute to a range of metabolic processes such as protein breakdown and carbohydrate fermentation [70].

LEfSe analysis revealed distinct microbial enrichment patterns in the rumen among the CON, AN, and CP groups (Figure 5). Specifically, the CON group showed significant enrichment of several taxa belonging to the *Clostridia_vadinBB60_group*, including unclassified members at the family and genus level. Notably, *Clostridia_vadinBB60_group* has been reported as a class of opportunistic pathogens linked to host metabolic dysregulation, with its abundance positively correlated with elevated fasting insulin levels and insulin resistance in various animal models [71]. In the present study, dietary supplementation with *Aspergillus niger* or the compound preparation reduced the relative abundance of these potentially detrimental bacteria, suggesting a role in supporting rumen health and metabolic homeostasis in Holstein bulls.

The unclassified genus *Prevotellaceae_g_unclassified* belongs to the family *Prevotellaceae* within the phylum *Bacteroidota*. Members of *Prevotellaceae* are widely recognized for their roles in degrading fibrous substrates and volatile fatty acids, and their abundance is strongly influenced by dietary composition. Previous studies have shown that higher dietary fiber content promotes the proliferation of *Prevotellaceae* in the gastrointestinal tract [72].

*Alloprevotella*, which was enriched in the AN group, is considered an early colonizer and core member of the gut microbiota. Its abundance is strongly influenced by dietary composition, and it contributes to gut health through the production of short-chain fatty acids, which serve as important signaling molecules and energy sources for intestinal epithelial cells.

*Alistipes*, a genus within the phylum *Bacteroidota*, facilitates the degradation of cellulose and other plant polysaccharides, thereby contributing to volatile fatty acid (VFA) synthesis. By supporting a stable rumen environment and microbial ecosystem, this genus may also indirectly enhance host health and production performance [73,74].

The differentially abundant taxa enriched in the CP group—*Acholeplasmatales*, *Acholeplasmataceae*, and *Anaeroplasma*—all belong to the phylum *Firmicutes*. As a dominant phylum in the rumen, *Firmicutes* play key roles in degrading cellulose and other plant polysaccharides, synthesizing fatty acids, and breaking down diverse organic substrates such as lipids, proteins, and carbohydrates [75]. Thus, supplementation with the compound preparation may enhance the degradation of dietary fiber and other nutrients. Nevertheless, the specific functional contributions of *Acholeplasmatales*, *Acholeplasmataceae*, and *Anaeroplasma* remain poorly characterized and merit further investigation.

LEfSe analysis of the fecal microbiota indicated no significantly enriched microbial taxa in the AN group, while one taxon was enriched in the CP group and three were enriched in the CON group. The differentially enriched microbe identified in the CP group, *Ruminococcaceae_g_uncultured*, belongs to the phylum *Firmicutes* and is phylogenetically affiliated with the genus *Ruminococcus*, a well-known fibrolytic bacterial group [68]. These results suggest that dietary supplementation with the compound preparation may enhance fiber digestion in the hindgut, though this potential effect requires further experimental validation.

The taxa *Saccharofermentans* and *Hungateiclostridiaceae*, which were enriched in the CON group, belong to the class *Clostridia* within the phylum *Firmicutes*. As Gram-positive, anaerobic fermentative bacteria, *Clostridia* are commonly found in environments such as soil and the animal intestinal tract. Although a small number of species are pathogenic, most function as commensal members of the gut microbiota. The observed reduction in the abundance of these taxa following supplementation with *Aspergillus niger* or its compound preparation suggests a potential suppressive effect on certain clostridial groups. However, the underlying mechanisms responsible for this shift remain to be fully elucidated.

Dietary supplementation with *Aspergillus niger* or its compound preparation preserved the dominant phyla in both the ruminal and fecal microbiota. Analysis of differentially abundant microbes indicated that these additives reduced the abundance of potentially detrimental taxa such as *Clostridia_vadinBB60_group*, while enriching beneficial genera including *Alloprevotella*, *Alistipes*, *Acholeplasmatales*, *Acholeplasmataceae*, and *Anaeroplasma*. These compositional shifts likely contributed to enhanced fiber degradation capacity and improved host health by modulating the functional structure of the rumen microbial ecosystem.

## 5. Conclusions

Dietary supplementation with *Aspergillus niger* or its compound preparation yielded three key benefits: mitigating heat stress, reducing methane emissions, and optimizing rumen function. Specifically, the supplements enhanced systemic antioxidant capacity, as evidenced by significantly elevated superoxide dismutase (SOD) activity in both supplemented groups and increased total antioxidant capacity (T-AOC) in the *Aspergillus niger* group. A numerical reduction in plasma HSP70 levels was also observed. These changes collectively alleviated heat-induced oxidative stress. The supplements also significantly reduced total enteric methane emissions, methane yield per unit body weight, and per-unit dry matter intake, highlighting their potential as effective methane-inhibiting feed additives. Although the overall structure of the rumen and fecal microbiota remained stable, the additives selectively modulated specific microbial populations: reducing the abundance of opportunistic pathogens such as *Clostridia_vadinBB60_group* while increasing fiber-degrading taxa like *Alloprevotella*, *Alistipes*, and *Acholeplasmatales*. These microbial shifts likely contributed to enhanced fiber digestion and improved host health status.

## Figures and Tables

**Table 1:** TMR raw material composition and nutrient level (DM basis).

Ingredient	Content	Nutritional Level ^(1)^	Content
Whole maize silage (%)	67.5	CP (%)	14.66
Wheat straw (%)	10	EE (%)	3.44
Concentrate (%)	22.5	NDF (%)	40.46
Ratio of concentrate to roughage	22.5:77.5	ADF (%)	23.08
		Ca (%)	1.21
		P (%)	0.54

^(1)^ Nutrient levels were measured values.

**Table 2:** Composition and nutritional level of concentrate raw materials (DM basis).

Ingredient	Content (%)	Nutrient Level	Content (%)
Corn	65.00	CP	20.58
Corn germ meal	11.50	EE	5.44
Sprayed corn	14.50	NDF	21.18
Limestone powder	1.00	ADF	11.05
Urea	0.75	Ca	1.06
NaHCO_3_	1.25	P	0.67
NaCl	1.00		
Premix ^(2)^	5.00		
Total	100.00		

^(2)^ The premix contained per kg DM: Fe 280 mg; Zn 1900 mg; Cu 350 mg; Mn 1900 mg; I 28 mg; Co 28 mg; VA 240,000 IU; VD3 84,000 IU; VE 300 mg.

**Table 3:** Effects of *Aspergillus niger* and compound preparation on respiratory rate and rectal temperature.

Items	Group			SEM	*p*-Value
	CON	CP	AN		
Rectal Temperature (°C)	38.64 ^a^	38.48 ^b^	38.40 ^b^	0.04	0.010
Respiratory Rate (/min)	70.46	71.67	64.34	2.11	0.328

Note: Different letters in the same row means significant difference (*p* < 0.05); The same letters in the same row means not significant difference (*p* > 0.05).

**Table 4:** Effects of *Aspergillus niger* and compound preparation on antioxidant capacity.

Items	Group			SEM	*p*-Value
	CON	CP	AN		
T-AOC (U/mL)	0.21 ^b^	0.23 ^b^	0.29 ^a^	0.01	0.016
SOD (U/mL)	10.28 ^b^	11.17 ^a^	11.30 ^a^	0.16	0.008
CAT (U/mL)	8.92	8.30	11.03	0.84	0.389
MDA (nmol/mL)	1.47	1.75	1.99	0.10	0.078

Note: Different letters in the same row means significant difference (*p* < 0.05); The same letters in the same row means not significant difference (*p* > 0.05).

**Table 5:** Effects of *Aspergillus niger* and compound preparation on VFA.

Items	Group			SEM	*p*-Value
	CON	CP	AN		
TVFA/(mmol/L)	46.68 ^b^	46.21 ^b^	55.95 ^a^	1.93	0.044
Acetate/(mmol/L)	32.67 ^b^	32.09 ^b^	38.67 ^a^	1.33	0.059
Propionate/(mmol/L)	8.45 ^b^	8.52 ^b^	10.32 ^a^	0.34	0.020
Butyrate/(mmol/L)	4.81	4.89	5.89	0.24	0.099
Isobutyrate/(mmol/L)	0.27 ^b^	0.27 ^b^	0.43 ^a^	0.02	0.001
Isovalerate/(mmol/L)	0.25	0.27	0.32	0.01	0.088
Acetate/Propionate	3.86	3.77	3.74	0.04	0.448

Note: Different letters in the same row means significant difference (*p* < 0.05); The same letters in the same row means not significant difference (*p* > 0.05).

**Table 6:** Effects of *Aspergillus niger* and complex microorganisms on gastrointestinal methane emissions.

Items	Group			SEM	*p*-Value
	CON	CP	AN		
Methane emission (L/d)	153.73 ^a^	71.25 ^b^	89.98 ^b^	11.20	0.002
Methane emission per unit of body weight (L/d/kg)	0.39 ^a^	0.18 ^b^	0.23 ^b^	0.03	0.002
Methane emission per unit of DMI (L/d/kg)	11.50 ^a^	5.56 ^b^	7.02 ^b^	1.08	0.003

Note: Different letters in the same row means significant difference (*p* < 0.05); The same letters in the same row means not significant difference (*p* > 0.05).

## Data Availability

The datasets generated and analyzed during the current study are not publicly available due to ongoing research based on the dataset but are available from the corresponding author on reasonable request.

## Supplementary Materials

The following supporting information can be downloaded at: https://www.mdpi.com/article/10.3390/ani16020154/s1, Table S1: Detailed data of sulfur hexafluoride (SF_6_) permeation tubes; Table S2: Plasma biochemical parameters not significantly affected by dietary treatments in heat-stressed Holstein bulls; Table S3: Detailed alpha diversity indices of the ruminal and fecal microbiota in Holstein bulls; Figure S1: Variation of the temperature-humidity index (THI) in the experimental barn across the trial period.

## Abbreviations

The following abbreviations are used in this manuscript:

AN	*Aspergillus niger* group
CP	Compound preparation group
CON	Control group
CH_4_	Methane
CO_2_	Carbon dioxide
SF_6_	Sulfur hexafluoride
THI	Temperature-Humidity Index
DMI	Dry matter intake
TMR	Total mixed ration
VFA	Volatile fatty acids
T-AOC	Total antioxidant capacity
SOD	Superoxide dismutase
CAT	Catalase
MDA	Malondialdehyde
HSP	Heat shock protein
Ig	Immunoglobulin
NH_3_-N	Ammonia nitrogen
MCP	Microbial crude protein
OUT	Operational taxonomic unit
PCoA	Principal coordinates analysis
LEfSe	Linear discriminant analysis Effect Size
LDA	Linear discriminant analysis
ANOVA	Analysis of variance
SEM	Standard error of the mean
