# Supplementary material for: Effects of Aspergillus niger and Its Compound Preparations on Methane Emissions and Gastrointestinal Microbiota in Heat-Stressed Holstein Bulls
Source: Animals (Basel). 2026 Jan 6;16(2):154. doi: 10.3390/ani16020154 (PMC12837928; doi:10.3390/ani16020154)

Article

Effects of *Aspergillus niger* and Its Compound Preparations on Methane Emissions and Gastrointestinal Microbiota in Heat-Stressed Holstein Bulls

Jiangge Wang, Shuaiqi Fu, Liyang Zhang, Xian Liu, Xianghui Yin, Shiqin Sun and Tengyun Gao *

Henan International Joint Laboratory of Nutrition Regulation and Ecological Raising of Domestic Animal, College of Animal Science and Technology, Henan Agricultural University, Zhengzhou 450046, China; wangjge2021@163.com (W.J.); fushuaiqi0371@163.com (F.S.); zhangliyang91@163.com(Z.L.);liuxian641@163.com(L.X.); coolboymommo@outlook.com (Y.X.); 13223861935@163.com (S.S.)

* Correspondence: dairycow@163.com

Supplementary Materials

Supplementary Table S1. Detailed data of sulfur hexafluoride (SF_6) permeation tubes.

Part A. Permeation tube data

Permeation Tube Number	Empty permeation tube weight (g)	Weight of pipe after filling with SF6 (g)	SF_6(g)	Remaining SF6 weight at deployment (g)
15	34.8302	36.0277	1.1975	1.0976
18	34.7484	35.9532	1.2048	1.0883
26	34.8063	36.1367	1.3304	1.2748
58	34.8788	36.2527	1.3739	1.2965
63	35.5206	36.7780	1.2574	1.0318
65	35.6002	37.2367	1.6365	1.5113
71	35.4345	36.8560	1.4215	1.2440
76	35.2761	36.7279	1.4518	1.2893
98	34.6566	36.1726	1.5160	1.2116
99	34.9457	36.2961	1.3504	1.2747
101	34.3682	35.8033	1.4351	1.2546
106	34.8267	36.1838	1.3571	1.0746
110	34.7798	36.2460	1.4662	1.2408
116	34.5725	35.8753	1.3028	1.2624
120	34.6987	36.3978	1.6991	1.4741
134	34.7204	36.2123	1.4919	1.3372
152	34.8021	35.9785	1.1764	1.1041
162	34.9901	36.2771	1.2870	1.2247
165	34.7764	36.0660	1.2896	1.1910
168	34.7894	36.5219	1.7325	1.6595
171	34.6651	36.0349	1.3698	1.3144
172	34.6967	36.1930	1.4963	1.2634
175	34.7721	36.4652	1.6931	1.3477
176	34.5076	35.4018	0.8942	0.8185

Part B. Permeation rate regression equations

Permeation Tube Number	Regression Equation	R^2	Permeation rate（mg/d）	Cattle Number
15	y=-0.00202 x + 36.0089	0.9997	2.02	C5
18	y=-0.00236 x + 35.9309	0.9997	2.36	C6
26	y=-0.00112 x + 36.1256	0.9995	1.12	B1
58	y=-0.00145 x + 36.2333	0.9997	1.45	C2
63	y=-0.00404 x + 36.7133	0.9996	4.04	B4
65	y=-0.00229 x + 37.2045	0.9983	2.29	B6
71	y=-0.00344 x + 36.8153	0.9993	3.44	C8
76	y=-0.00278 x + 36.6758	0.9994	2.78	A7
98	y=-0.00526 x + 36.0776	0.9996	5.26	C3
99	y=-0.00163 x + 36.2858	0.9992	1.63	A2
101	y=-0.00357 x + 35.7654	0.9997	3.57	B7
106	y=-0.00491 x + 36.0965	0.9996	4.91	A8
110	y=-0.00391 x + 36.1761	0.9996	3.91	B5
116	y=-0.00082 x + 35.8675	0.9991	0.82	A4
120	y=-0.00380 x + 36.3237	0.9995	3.80	A3
134	y=-0.00272 x + 36.1665	0.9997	2.72	A6
152	y=-0.00144 x + 35.9633	0.9995	1.44	A5
162	y=-0.00111 x + 36.2592	0.9995	1.11	B2
165	y=-0.00191 x + 36.0434	0.9994	1.91	B8
168	y=-0.00127 x + 36.4992	0.9992	1.27	A1
171	y=-0.00110 x + 36.0234	0.9993	1.10	C1
172	y=-0.00400 x + 36.1187	0.9994	4.00	B3
175	y=-0.00588 x + 36.3532	0.9993	5.88	C7
176	y=-0.00129 x + 35.3774	0.9995	1.29	C4

Supplementary Table S2. Plasma biochemical parameters not significantly affected by dietary treatments in heat-stressed Holstein bulls.

Data are presented as mean ± standard error of the mean (SEM). *P*-values are from one-way ANOVA; CON, control group; CP, compound preparation group; AN, *Aspergillus niger* group.

Section A. Heat shock proteins

Items	Group			SEM	*P*-value
	CON	CP	AN		
HSP70（pg/mL）	2600.25	2396.44	2484.68	39.97	0.109
HSP90（pg/mL）	2211.00	2051.19	2165.18	36.84	0.195

Section B. Hormones

Items	Group			SEM	*P*-value
	CON	CP	AN		
T-AOC（U/mL）	0.21^b	0.23^b	0.29^a	0.01	0.016
SOD（U/mL）	10.28^b	11.17^a	11.30^a	0.16	0.008
CAT（U/mL）	8.92	8.30	11.03	0.84	0.389
MDA（nmol/mL）	1.47	1.75	1.99	0.10	0.078

Section C. Immunoglobulins

Items	Group			SEM	*P*-value
	CON	CP	AN		
IgG（mg/mL）	18.42	17.14	19.27	0.40	0.074
IgA（μg/mL）	228.54	228.63	228.43	3.45	1.000
IgM（μg/mL）	535.36	560.31	551.67	10.00	0.619

Supplementary Table S3. Detailed alpha diversity indices of the ruminal and fecal microbiota in Holstein bulls.

Data are presented as mean. SEM, standard error of the mean. CON, control group; CP, compound preparation group; AN, *Aspergillus niger* group. No significant differences were observed among groups for any index (P > 0.05).

Part A. Rumen microbiota

**Items**	**Group**			**SEM**	***P*-value**
	**CON**	**CP**	**AN**		
Reads	36958.14	35361.38	35186.38	505.14	0.319
OTU	1738.57	1700.88	1598.38	29.40	0.131
Ace	2068.71	2014.63	1916.75	29.17	0.096
Chao	2091.71	2036.38	1951.50	28.72	0.136
Coverage	0.9891	0.9891	0.9892	0.0002	0.956
Shannon	5.8629	5.7950	5.7525	0.0332	0.424
Simpson	0.0122	0.0158	0.0126	0.0010	0.304

Part B. Fecal microbiota

**Items**	**Group**			**SEM**	***P*-value**
	**CON**	**CP**	**AN**		
Reads	36958.14	35361.38	35186.38	505.14	0.319
OTU	1738.57	1700.88	1598.38	29.40	0.131
Ace	2068.71	2014.63	1916.75	29.17	0.096
Chao	2091.71	2036.38	1951.50	28.72	0.136
Coverage	0.9891	0.9891	0.9892	0.0002	0.956
Shannon	5.8629	5.7950	5.7525	0.0332	0.424
Simpson	0.0122	0.0158	0.0126	0.0010	0.304

Supplementary Figure S1. Variation of the temperature-humidity index (THI) in the experimental barn across the trial period.

The black solid line indicates the heat stress threshold (THI = 68). The index consistently remained above this threshold, confirming that the bulls were subjected to continuous moderate-to-severe heat stress throughout the study.

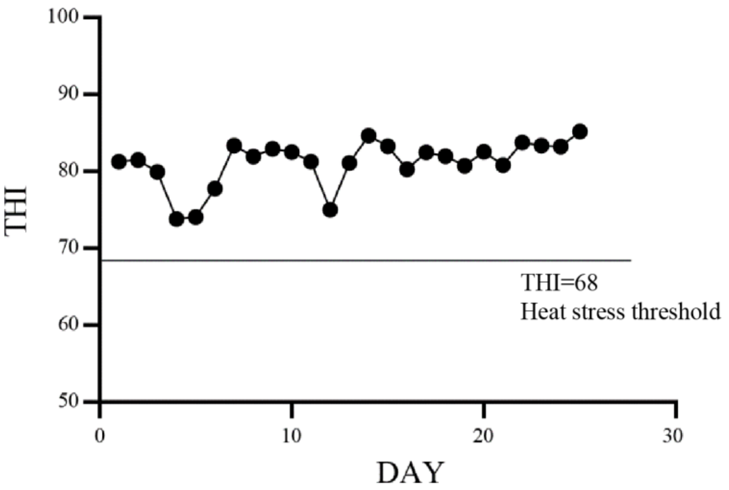

Supplement: Supplementary file 1 [file animals-16-00154-s001.zip › animals-4010171-supplementary.pdf]
